# Comparison of the effect of a combination of eight micronutrients versus a standard mono preparation on sperm parameters

**DOI:** 10.1186/s12958-016-0219-0

**Published:** 2016-12-09

**Authors:** Markus Lipovac, Florian Bodner, Martin Imhof, Peter Chedraui

**Affiliations:** 1 IMI Fertility Center, Vienna, Austria; 2 Karl Landsteiner Institute for cell-based therapy in Gynecology, Wiener Ring 3-5, 2100 Korneuburg, Austria; 3 Medical University of Vienna, Vienna, Austria; 4 Institute of Biomedicine, Research Area for Women’s Health, Facultad de Ciencias Médicas, Universidad Católica de Santiago de Guayaquil, Guayaquil, Ecuador

**Keywords:** Sperm parameters, Combined treatment, Mono-treatment, Micronutrients, l-carnitine

## Abstract

**Background:**

There are reports showing that l-carnitine alone or in combination with other micronutrients improve sperm parameters. However, comparative studies are still lacking. This study was carried out to compare the short term effects of a combination of eight micronutrients including l-carnitine vs. a mono-substance (l-carnitine alone) on sperm parameters.

**Methods:**

This was a prospective, open-labelled, nonrandomized study that included male subjects (20 to 60 years) with at least 1 year of subfertility and at least one pathological semen analysis who received 3 months treatment with a mono-substance (500 mg l-carnitine/twice a day, *n* = 156) or a combined compound (440 mg l-carnitine + 250 mg l-arginine + 40 mg zinc + 120 mg vitamin E + 80 mg glutathione + 60 μg selenium + 15 mg coenzyme Q10 + 800 μg folic acid/once a day, *n* = 143) for the same time period. Sperm parameters were analyzed before and after treatment and groups comparisons performed.

**Results:**

Baseline characteristics were similar among studied groups (age and body mass indices). Semen parameters (volume, density, overall progressive motility [including slow and fast motility]) and percentage of sperm with normal morphology improved after 3 months in both groups as compared to baseline. However, relative change (expressed as % increase of absolute values) for sperm density and overall progressive motility (including fast motility) was found to be higher for the combined micronutrient treatment group as compared to the mono-treatment using l-carnitine alone.

**Conclusion:**

Both analyzed groups displayed a positive short term effect on all sperm parameters; however effect on density and motility was significantly better for the combined formulation. There is need for more research in this matter that includes long term outcome data.

**Trial registration:**

Retrospectively registered at ISRCTN (7th October 2016). Study ID: ISRCTN48594239

## Background

At the peak of reproductive capability, a healthy couple has an annual pregnancy probability rate of 80–85%. Nevertheless, 10–15% of couples trying to achieve pregnancy remain childless [1]. Male factor accounts for nearly half of cases of infertility presenting an abnormal semen analysis. In 45% of sub-/infertile men, this disorder is of unexplained (unidentified) origin [2, 3].

Reports indicate that sperm quality has declined over the past decades [4, 5]. Mainly, this fact seems to be the result of certain nutritional deficiencies and adverse environmental and lifestyle factors [6, 7]. Various micronutrients [8–11] are directly or indirectly involved in sperm metabolism; and nutrient deficiencies can lead to impaired sperm quality [12, 13]. Ionising radiation, genital infections and alcohol, cigarette or illicit drug consumption can cause intrinsic toxicity and generate intense body oxidative stress. The negative impact of reactive oxygen species (ROS) has been recognized as a main cause of idiopathic infertility [14, 15]. Free radicals damage spermatic nuclear and mitochondrial genetic information producing strand breaks in the DNA [16, 17]; thus, impairing their mobility and morphology through lipid peroxidation of the plasma membrane, and compromising sperm cell and oocyte interaction [18–20].

In a Cochrane review, it has been reported that antioxidant supplementation in sub-fertile males may improve the outcomes of live birth and pregnancy rate for sub-fertile couples undergoing ART cycles [17]. The suspected mechanism of action is the improvement of sperm quality through the alleviation of the nutrient deficiency as well as the reduction of oxidative stress [21]. The sum of these effects leads to a higher chance of pregnancy, an undisturbed intrauterine development and a certain inherent protection of the newborn [22–33].

Previous studies have assessed the use of l-carnitine alone as well as various combination therapies as an effective way of improving semen analysis results [34–42]. However, a direct contrast with a combination of various micronutrients and, consequently, a quantification of the effects, has not yet been assessed in the framework of a research study. Hence, the aim of the present investigation was to compare the short term effect of a combination of eight micronutrients (including l-carnitine) vs. a standard mono-substance (l-carnitine alone) on sperm parameters.

## Methods

This was a prospective, open-labelled, non-randomized study that included male subjects consulting according to inclusion criteria between January 2005 and January 2014 at the IMI Fertility Center and the Med19 Study Center of Vienna, Austria.

A total of 299 participants who met inclusion criteria: 20 to 60 years of age, suffering from at least 1 year of subfertility and had at least one recent pathological semen analysis result (up to 3 months, considered baseline) were invited to participate for 3 months to receive treatment with a mono-substance (500 mg l-carnitine/twice a day, *n* = 156) or a combined compound (440 mg l-carnitine + 250 mg l-arginine + 40 mg zinc + 120 mg vitamin E + 80 mg glutathione + 60 μg selenium + 15 mg coenzyme Q10 + 800 μg folic acid/once a day, *n* = 143; Profertil®, Lenus Pharma GmbH, Vienna, Austria) for the same period of time. A second semen analysis was performed after 3 months. Subjects were encouraged to refrain from the consumption of other micronutrients during the study period. Exclusion criteria were: azoospermia, aspermia, varicocele, recent urogenital infections, and hormonal disorders.

The following sperm parameters were analysed at baseline and after treatment: sperm volume (ml) and density (mio/ml), overall, fast and slow progressive motility (expressed as %), and % of sperm with normal morphology. All parameters were defined according to WHO guidelines (4th edition). Local ethical approval was obtained for this study which was retrospectively registered at ISRCTN (7th October 2016). Study ID: ISRCTN48594239 www.isrctn.com. All subjects were informed of the research, its objectives and provided signed consent of participation and publication of individual patient data. Pseudonymisation and access restrictions were used in order to avoid public access to the sensitive patient data.

Statistical analysis was performed with the Statistical Package for the Social Sciences version 22.0 (IBM SPSS, Armonk, NY, USA). All data are presented as mean ± standard deviations. The Kolmogorov Smirnov test was used to assess the normality of data distribution. For each measured variable and each case changes from baseline (increase, decrease or none) were calculated as absolute (subtraction of 3rd month value minus baseline) or relative values (the percent expression of each calculated absolute value). Paired Student’s *T* test was used for intragroup comparisons. Non paired Student’s *T* test or the Mann Whitney *U* test was used for intergroup comparisons. A *p* value of <0.05 was considered as statistically significant.

## Results

Age ranged from 20 to 60 years in both groups, displaying similar median age and body mass indices. Other baseline characteristics such alcohol and tobacco consumption were similar among studied groups. The effect of each treatment on semen parameters after 3 months is presented in Table 1. It was observed that all studied sperm parameters (volume, density, overall progressive motility [including slow and fast motility]) and % of sperm with normal morphology significantly improved after 3 months of treatment in both groups as compared to baseline. However, relative change (expressed as % increase of absolute values) for sperm density and overall progressive motility (including fast motility) was found to be higher for the combined micronutrient treatment group as compared to the mono-treatment group using l-carnitine alone. Worth to note was the fact that the baseline slow- and fast progressive motility and % of normal morphology were significantly different between studied groups.

**Table 1:** Semen parameters before and after 3 months according to each treatment group

	Mono treatment *n* = 156				Combined treatment *n* = 143			
	Basal	3 months	Absolute change^a^	Relative change (%) ^b^	Basal	3 months	Absolute change^a^	Relative change (%) ^b^
Sperm volume (ml)	3.0 ± 1.2	3.5 ± 1.5^*^	0.5 ± 1.6	35.2 ± 75.3	2.9 ± 1.2	3.6 ± 1.4^*^	0.7 ± 1.4	41.5 ± 74.3
Sperm density (mio/ml)	28.5 ± 19.0	34.2 ± 21.7^*^	5.7 ± 21.3	63.7 ± 158.0	24.9 ± 16.2	32.8 ± 20.0^*^	7.9 ± 23.5	157.7 ± 400.4^**^
Overall progressive motility (%)	35.7 ± 15.8	44.9 ± 19.9^*^	9.2 ± 18.6	44.0 ± 90.3	33.4 ± 14.6	48.6 ± 17.6^*^	15.2 ± 17.9^**^	80.3 ± 142.5^**^
*fast progressive motility (%)*	16.7 ± 10.6	23.0 ± 14.0^*^	6.3 ± 16.0	87.4 ± 184.0	10.3 ± 9.4^**^	20.4 ± 13.1^*^	10.1 ± 15.0^**^	259.3 ± 389.1^**^
*slow progressive motility (%)*	19.2 ± 12.7	22.2 ± 13.1^*^	2.9 ± 12.8	125.9 ± 384.9	23.1 ± 12.9^**^	28.3 ± 12.2^*, **^	5.1 ± 12.7	102.9 ± 261.5
Normal morphology (%)	17.9 ± 14.0	27.5 ± 16.1^*^	9.6 ± 17.8	230.3 ± 668.1	26.2 ± 15.6^**^	35.9 ± 16.1^*, **^	9.7 ± 19.4	307.1 ± 1,012.2

Data are presented as mean ± standard deviations

^*^ *p* value < 0.001 as compared to basal as determined with the paired Student’s *T* test

^**^ *p* value < 0.05 as compared to mono treatment as determined with the non paired Student’s *T* test

^a^ Absolute change for each variable and case was calculated subtracting third month value minus basal one

^b^ relative change is the percent expression of each calculated absolute value

## Discussion

The effect of the administered mono-substance composed of the hydrophile molecule l-carnitine can be ascribed to its properties as a radical catcher in the mitochondrial membrane. By substitution anti-oxidative capacity in the seminal plasma increases significantly [43]. Moreover, studies in animals have shown that the molecule has protective effects on testicular tissue which has been exposed to ionising radiation [44, 45]. This realisation can have a relevant effect on sperm quality in certain daily situations. Both effects serve as an explanation model for the positive influence of l-carnitine on sperm concentration, motility and morphology [34, 46, 47]. Consistent with these studies, our study found that l-carnitine alone or in combination with seven other micronutrients displayed short term improvement of all studied sperm parameters. Nevertheless, the combined preparation showed better results through a higher percent increase for sperm density and overall progressive motility (including fast motility) as compared to l-carnitine alone. To the best of our knowledge this research may be the first to prospectively compare the short-term effects of a mono treatment versus a combined product on sperm parameters.

The effect of the combination substance is to be seen as the sum of the partly augmentative effects of each component. The water-soluble l-arginine has a positive effect on sperm parameters such as motility and vitality [48, 49]. The hydrophile coenzyme Q10 catches free radicals, and improves male FSH and LH serum profile [9, 50] and shows an improvement over sperm concentration, motility and morphology [9, 51]. The hydrophile dietary mineral zinc provides a protective antioxidant effect during sperm production that may counteract against copper excess [52, 53], improving sperm cell concentration and motility [54–57]. Hydrophile folic acid reduces the concentration of round cells in the semen which are a major source for free radicals [58, 59]; and hence leading to a reduction of oxidative stress [60] as well as a rise in sperm cell concentration [61]. Furthermore, there is evidence that folic acid could compensate for adverse influences of hypothyroidism and thyrostatic substances on testicular tissue [62, 63]. Selenium reduces oxidative stress [64] as it is bound as a structural element in the sperm cell membrane in form of the enzymes selenoprotein mGPx4 and snGPx4 [65]. Moreover, it optimizes thyroid metabolism important for sperm production and has a positive influence on autoimmune processes [66]. Selenium intake improves sperm motility [67] and morphology [65]. The water-soluble glutathione reduces oxidative stress significantly [68] which leads to an increase of progressive and morphologically normal sperm cells [38]. Regarding oxidative stress, one study found that l-carnitine improved sperm motility in asthenozoospermic men with normal levels of phospholipid hydroperoxide glutathione peroxidase, an important enzyme that minimizes oxidative stress [69]. The liposoluble vitamin E as component of the human cell membrane acts as oxidative protector for likewise located unsaturated fatty acids [70]. Vitamin E supplementation reduces lipid peroxidation, therefore improving sperm motility significantly [21]. This antioxidative effect may potentiate the beneficial effects of l-carnitine. Indeed, one study found that the combination of l-carnitine (2 g per day) and vitamin E improved sperm motility in asthenozoospermic men; effect which was not observed among those taking vitamin E alone [71].

L-carnitine seems not the sole actor in sperm production. As already mentioned, the combined compound displayed a better improvement of sperm density and overall progressive motility. This is important because motility seems to be a key parameter of male fertility. The diverse target points for eight micronutrients and their partially synergistic cooperation in different body compartments serve as an explanatory model for its selective superiority as compared to the mono-therapy with l-carnitine alone. It is still unclear whether motility improvement exerted by combined compound is due to enhanced oxidative stress suppression or to the sum of the specific singular effects of each compound.

Authors acknowledge that the study has limitations. Indeed, although data is prospective, its open-labelled and non-randomized nature is a potential source of bias. On the other hand, this document does not present final outcome of pregnancies; however, this was not the primary endpoint aim of this study arm. Data related to outcome of pregnancy, as a secondary end-point, will be presented elsewhere.

Despite these limitations, prospective comparative data regarding the efficacy of mono-treatment and combined compound on sperm parameters are lacking. Hence, this may be seen as a potential strength of our study. Nevertheless, there is need for well controlled prospective randomized data.

## Conclusion

Overall effect on sperm parameters was similar in both analyzed groups; however, in the combined-formulation group improvement of sperm density, overall and fast progressive motility was significantly better than those in the mono-preparation group. There is a need for more research in this matter that include pregnancy outcome data.

## Abbreviations

ART: Assisted reproductive technology
ROS: Reactive oxygen species
WHO: World Health Organization
